# Factors associated with preference for in-person versus remote consultations in inflammatory bowel disease in the United Kingdom: secondary analysis of an online survey

**DOI:** 10.1097/MEG.0000000000003220

**Published:** 2026-06-04

**Authors:** Keith Bodger, Frederick Taylor, Pratik Rai, Liz Dobson, Judith Bunn, Stephen Grainger, Fraser Cummings, Stuart Bloom, Nicholas Kennedy

**Affiliations:** a Department of Health Data Science, University of Liverpool; b Department of Gastroenterology, Liverpool University Hospitals NHS Foundation Trust, Liverpool; c IBD Registry, London; d Department of Gastroenterology, University Hospital Southampton, Southampton; e Department of Gastroenterology, University College London Hospitals NHS Foundation Trust, London; f Department of Gastroenterology, Royal Devon & Exeter NHS Foundation Trust, Exeter, UK

**Keywords:** inflammatory bowel diseases, patient preference, socioeconomic factors, surveys and questionnaires, telemedicine

## Abstract

**Introduction:**

The coronavirus disease 2019 pandemic accelerated use of remote consultations as an alternative to in-person inflammatory bowel disease (IBD) care. However, factors associated with preference for remote or in-person follow-up remain poorly understood.

**Methods:**

We undertook a retrospective analysis of an electronic follow-up survey completed by patients who had previously used an online IBD self-assessment tool during the early pandemic. Respondents with prior experience of remote consultations were included. Preference for future consultation modality was categorised as strong in-person preference (‘Never by telephone or video’), strong remote preference (‘Mostly by telephone or video’), or no preference. Survey domains included sociodemographics, comorbidities, home restriction, mobility, support, IBD-Control Questionnaire, patient reported outcome‐2 symptoms, and satisfaction with IBD care. Associations with preference were examined using binary logistic regression and ordinal logistic regression across ordered preference categories from remote to no preference to in-person.

**Results:**

Among 5833 eligible respondents, 65% reported no preference, 25% a strong remote preference, and 10% a strong in-person preference. Stronger in-person preference was associated with older age, male gender, greater area deprivation, Asian ethnicity compared with White ethnicity, two or more comorbidities, Crohn’s disease compared with ulcerative colitis, poorer IBD-specific patient-reported outcome measures, higher patient reported outcome‐2 symptom scores, and dissatisfaction with care. Associations with strong remote preference were generally opposite, including younger age, lower deprivation, fewer comorbidities, ulcerative colitis, better patient reported outcome measure scores, and greater satisfaction; gender and ethnicity were not significant. Home restriction, mobility, and support were not significantly associated. Negative IBD-Control-8 responses increased progressively from remote preference to no preference to in-person preference.

**Conclusion:**

Sociodemographic and clinical factors influence consultation preferences in IBD. These findings may support equitable integration of remote care models.

## Introduction

Telephone or video consultations have become increasingly commonplace in healthcare and have reduced the traditional reliance of services on consultations delivered in-person at healthcare facilities [1]. Remote consultations offer potential benefits to both patients and providers in terms of better convenience at lower costs [2]. The coronavirus disease 2019 (COVID-19) pandemic accelerated the adoption of remote consultations across many conditions, including inflammatory bowel disease. Various telehealth interventions have been shown to offer similar outcomes to traditional in-person IBD care, at least among people willing to participate in trials of such care models [3]. However, concerns have been raised about the widespread adoption of remote consultations across healthcare and whether services have appropriate safeguards in place to ensure patient preferences are considered, and potential inequalities in access to care are avoided [4–6]. In the postpandemic period, it is unclear whether IBD services have reconfigured clinics to offer the right balance of in-person and remote appointments, nor whether patient preferences are adequately factored into the provision of appointments. Recent National Health Service policy in the UK strongly promotes greater adoption of remote services and reduced reliance on traditional in-person secondary care models [7].

A number of factors have been reported to be associated with patient preferences for modality of consultations in primary care [8,9] or in specific outpatient conditions [10–12]. However, there has been limited research focussed on people living with inflammatory bowel disease. Existing studies have often been small and restricted to single centres, thereby limiting their generalisability. Little is known about the relative importance of generic factors (e.g. age, gender, socioeconomic status, mobility, multimorbidity) as opposed to more disease-specific factors such as IBD subtype, symptoms, and disease-specific quality of life. A better understanding could inform refinements to the way services provide alternative modalities for consultations.

We report a secondary analysis of data from the IBD Registry COVID-19 follow-up survey, a large-scale UK-wide survey of IBD patients undertaken in 2021, which included questions about preferences for modality of future IBD consultations and information about sociodemographics, diagnosis, comorbidities, current health status, and experience of IBD care.

## Methods

### Survey

As we reported previously [13], the IBD Registry COVID-19 follow-up survey was distributed by e-mail between July and November 2021 to people who had originally completed an online self-assessment tool during the early phase of the pandemic and provided consent to be recontacted for future research.

The details of the survey questionnaire have been reported [13]. All items included in the present study are shown in Supplementary Materials (Appendix S1, Supplemental digital content 1, https://links.lww.com/EJGH/B315). We extracted data for age, gender, ethnicity, place of residence (to allow mapping to the relevant index of multiple deprivation), self-reported IBD diagnosis, and total number of comorbidities across seven possible categories (hypertension; respiratory or chest disease; angina, heart attack, or stroke; heart failure; heart valve disease; diabetes mellitus; chronic kidney disease). There were additional questions about whether the patient had any medical condition that required them to stay at home and any limitations to mobility. The survey included two IBD-specific patient-reported outcome measures [IBD-Control Questionnaire (IBD-Control-8 and IBD-Control-Visual Analogue Scale {VAS}) [14]; and patient reported outcome-2 (PRO-2) symptom-based score (Crohn’s disease-PRO-2 or ulcerative colitis-PRO-2), according to subtype] [13]. IBD-Control-8 measures ‘disease control from the patient perspective’, a construct that includes disease-specific quality of life (QoL) domains [i.e. physical (pain, fatigue, sleep), psychological (anxiety/depression), and social (missing planned activities)] and a treatment domain (perceived efficacy of current treatment, need for treatment change) [13,14].

In relation to the modality of IBD consultations, specific questions covered whether or not the patient had any past experience of receiving telephone or video consultations, and a single item asking about current preference for future consultations. There was a question about satisfaction with care, which asked whether respondents believed that their IBD care needs had been met over the course of the pandemic.

### Eligibility criteria

We included only patients who reported having had some prior experience of telephone or video consultation with their IBD service, thereby investigating preferences only among people who had direct experience of both in-person and remote care. Other inclusion criteria were: (a) Diagnosis of either ulcerative colitis or Crohn’s disease; (b) Complete data for the primary outcome (preference question) and for the main covariates included in baseline models (complete cases). Of the 40 911 patients invited to complete the COVID-19 follow-up, 7341 adults returned a response [13] and 5833 were eligible for the present analysis. Details of cohort selection and exclusions are shown in Fig. 1.

**Fig. 1.**
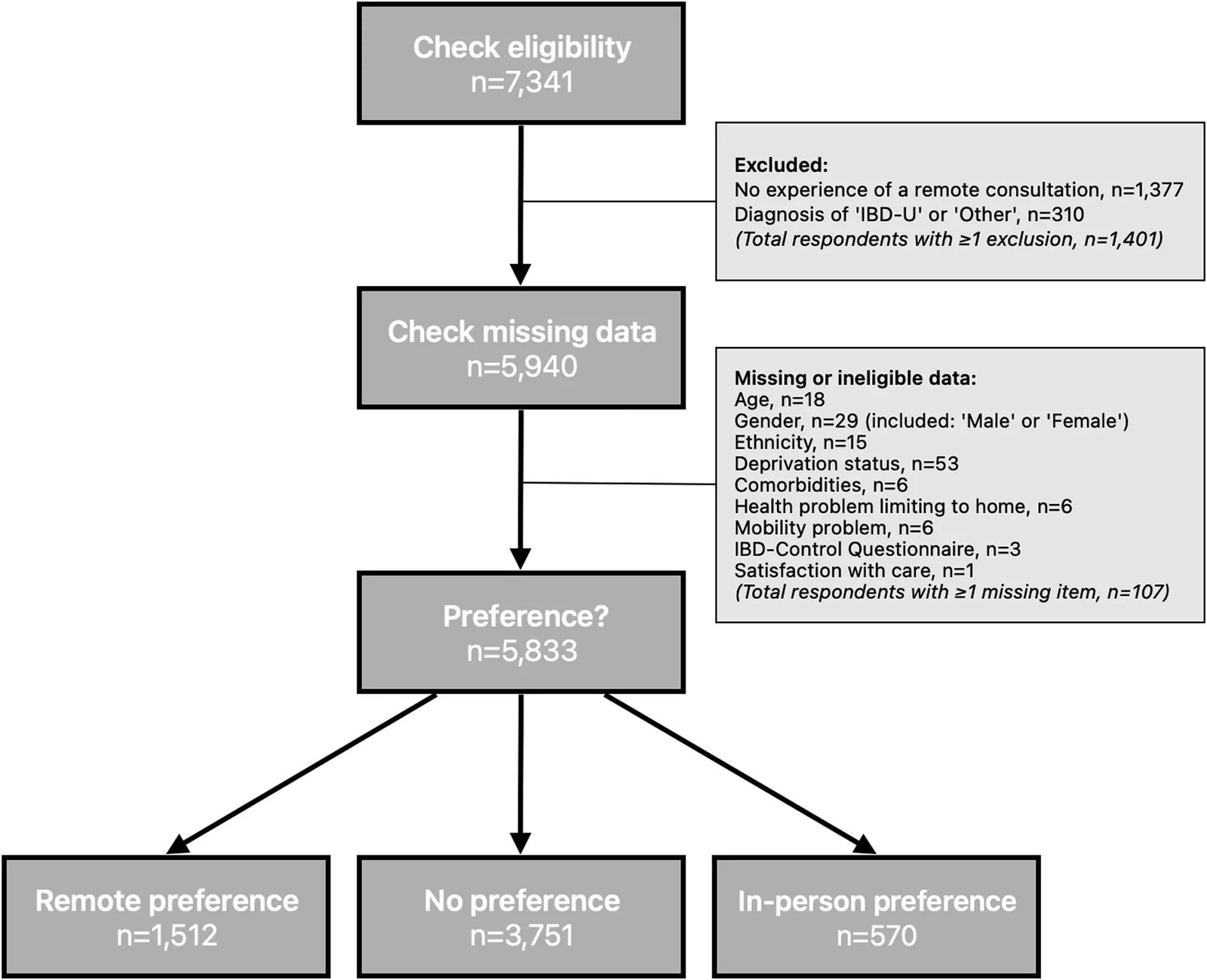
Flow diagram showing selection of cases and categorisation of cohort by current preference for modality of future IBD consultation. IBD, inflammatory bowel disease.

### Primary outcome

The primary outcome for this study was derived from a question about current preference for future IBD consultations: *‘What would be your preference for IBD appointments in the future?’*. Patients were categorised as having a strong remote preference if they selected ‘*mostly by telephone or video*’. Those who selected ‘*never by telephone or video*’ were categorised as having a strong in-person preference. The remainder were categorised as having no preference (‘*sometimes by telephone or video*’ or ‘*I do not mind*’).

### Potential predictors of preference for in-person versus remote consultations

On the basis of evidence from other conditions [8–12], we hypothesised that a number of generic factors may be associated with preference for in-person as opposed to remote consultations: age; gender; socioeconomic status; ethnicity, number of comorbid conditions, mobility problems or health conditions restricting to the home, satisfaction with previous care. In relation to IBD-specific factors, we postulated that patients with more severe disease at the time of completing the survey would favour in-person assessment, as reflected by lower scores for the IBD-Control Questionnaire or higher PRO-2 symptom-based scores. Conversely, better control or lower disease activity would be associated with an increased likelihood of remote preference. We further hypothesised that in-person preference might be associated with Crohn’s disease (relative to ulcerative colitis), because of its greater propensity for relevant physical signs (e.g. abdominal mass, fistulae) and complexity, which might influence how patients perceive the benefits of face-to-face assessments.

### Statistical analysis

Modelling of associations – To explore associations between patient characteristics and preference, we undertook complementary sets of analyses, including two sets of binary logistic regression (BLR) models and one set of ordinal logistic regression (OLR) models. The first set of BLR models explored ‘*What factors are associated with a strong in-person preference?*’ by comparing characteristics of people who selected ‘*Never by telephone or video*’ versus everyone else. The second set of BLR models explored ‘*What factors are associated with a strong remote preference?*’ by comparing those who selected ‘*Mostly by telephone or video*’ versus everyone else.

In the third set of analyses, we considered in-person preference as a three-level ordinal variable, using OLR to test associations. In contrast to the BLR, this approach considers in-person preference to be a spectrum, moving from remote through no preference to in-person, and considers the question ‘*What factors are associated with being in a higher category of in-person preference*’. The OLR model estimates the cumulative odds of being in a higher category – that is, In-person *versus* None or Remote; and In-person or None *versus* Remote. Results for all models are presented as unadjusted (i.e. univariate associations) and adjusted (i.e. multivariate) odds ratios (OR) with their 95% confidence intervals provided in relevant tables.

Study data were collected and managed using REDCap electronic data capture tools hosted at the IBD Registry Ltd. All analyses were performed using de-identified data extracted from REDCap to R (Version 4.1.0, R Core Team 2021, R Foundation for Statistical Computing, Vienna, Austria) using the *redcapAPI* package [15,16]. The ‘*MASS’* package was used for OLR and ‘*performance’* for model diagnostics.

## Results

### Patient characteristics

Table 1 shows the characteristics and survey responses of 5833 eligible respondents overall and stratified by preference category for the modality of consultation. Median age was 48 years, with 15% (*n* = 865) were aged more than 65 years. There were more female (64%) respondents than males (36%), and 96% reported White ethnicity. There were more patients with Crohn’s disease (55%) than ulcerative colitis (45%). One in four respondents reported at least one comorbidity, with 6% (*n* = 336) reporting greater than or equal to 2 conditions.

**Table 1.** Characteristics of 5833 survey respondents with prior experience of both in-person and remote consultations

Characteristic	Overall (*N *= 5833)	Remote preference (*N *= 1512)	No preference (*N* = 3751)	In-person preference (*N* = 570)
Age^a^	48 (37–59)	48 (37–58)	48 (37–59)	51 (37–62)
Age group (years)				
≤65	4968 (85%)	1311 (87%)	3197 (85%)	460 (81%)
>65	865 (15%)	201 (13%)	554 (15%)	110 (19%)
Gender				
Female	3723 (64%)	931 (62%)	2447 (65%)	345 (61%)
Male	2110 (36%)	581 (38%)	1304 (35%)	225 (39%)
Deprivation quintile				
1 – Least deprived	1585 (27%)	440 (29%)	1016 (27%)	129 (23%)
2	1433 (25%)	396 (26%)	906 (24%)	131 (23%)
3	1231 (21%)	322 (21%)	793 (21%)	116 (20%)
4	992 (17%)	226 (15%)	649 (17%)	117 (21%)
5 – Most deprived	592 (10%)	128 (9%)	387 (10%)	77 (14%)
Ethnicity				
White	5627 (96%)	1463 (97%)	3619 (96%)	545 (96%)
Asian	108 (1.9%)	21 (1.4%)	68 (1.8%)	19 (3.3%)
Black	15 (0.3%)	3 (0.2%)	11 (0.3%)	1 (0.2%)
Mixed	68 (1.2%)	19 (1.3%)	46 (1.2%)	3 (0.5%)
Any other	15 (0.3%)	6 (0.4%)	7 (0.2%)	2 (0.4%)
Diagnosis				
Ulcerative colitis	2607 (45%)	757 (50%)	1615 (43%)	235 (41%)
Crohn’s disease	3226 (55%)	755 (50%)	2136 (57%)	335 (59%)
IBD-Control-8^a^	12 (8, 15)	14 (10, 16)	12 (7, 15)	10.0 (6, 14)
IBD-Control-VAS^a^	80 (60, 93)	85 (70, 96)	80 (54, 92)	75 (50, 88)
PRO-2 remission				
Active	1580 (27%)	349 (23%)	1025 (27%)	206 (36%)
Remission	3886 (67%)	1110 (73%)	2456 (65%)	320 (56%)
* *Missing^b^	367 (6%)	53 (4%)	270 (7%)	44 (8%)
Comorbidities				
0	4385 (75%)	1157 (77%)	2829 (75%)	399 (70%)
1	1112 (19%)	283 (19%)	711 (19%)	118 (21%)
2+	336 (6%)	72 (4.8%)	211 (6%)	53 (9.3%)
Restriction to the home	752 (13%)	164 (11%)	501 (13%)	87 (15%)
Mobility problems	255 (4%)	54 (4%)	173 (5%)	28 (5%)
Support available	5110 (88%)	1337 (88%)	3288 (88%)	485 (85%)
Satisfied with care				
Yes, completely	3148 (54%)	1048 (69%)	1936 (52%)	164 (29%)
Yes, mostly	2196 (38%)	380 (25%)	1506 (40%)	310 (54%)
No, not at all	246 (4%)	40 (3%)	144 (4%)	62 (11%)
* *Missing^b^	243 (4%)	44 (3%)	165 (4%)	34 (6%)

Overall and stratified by preference for future IBD consultations.

IBD, inflammatory bowel disease; PRO-2, patient reported outcome-2; VAS, Visual Analogue Scale.

a Median (interquartile range).

b Cases with missing observations were excluded from relevant analyses.

### Preferences for modality of consultation

Ten percent (*n* = 570) of the eligible cohort expressed a strong preference for future in-person consultations (Response: ‘Never by telephone or video’), 65% (*n* = 3751) reported no preference, and 25% (*n* = 1512) favoured predominantly remote consultations (‘Mostly by telephone or video’).

### Factors associated with preference for the modality of consultation

Results of the complementary sets of BLR models are shown in Table 2. These examined factors associated with a strong preference for either in-person consultation (versus not), or for remote consultation (versus not), respectively. Findings from the OLR models are shown in Supplementary Table S1, Supplemental digital content 1, https://links.lww.com/EJGH/B315, which examined whether these same factors were associated with a ‘higher level’ or ‘increased degree’ of in-person preference. A comparison of key model findings across BLR and OLR models is shown in Supplementary Table S2, Supplemental digital content 1, https://links.lww.com/EJGH/B315.

**Table 2.** Factors associated with a strong preference for modality of future inflammatory bowel disease consultations among 5833 survey respondents with prior experience of both in-person and remote consultations:

Characteristic	In-person preference (versus not)^a^				Remote preference (versus not)^b^			
	OR	95% CI	aOR	95% CI	OR	95% CI	aOR	95% CI
Age group								
≤65 Years (ref)	–	–	–	–	–	–	–	–
>65 Years	1.43	1.14–1.78	**1.59**	1.24–2.03	0.84	0.71–1.00	**0.71**	0.59–0.85
Gender								
Female (ref)	–	–	–	–	–	–	–	–
Male	1.17	0.98–1.39	**1.59**	1.24–2.03	1.14	1.01–1.29	1.04	0.91–1.18
Deprivation quintile								
1 – Least deprived (ref)	–	–	–	–	–	–	–	–
2	1.14	0.88–1.47	1.09	0.84–1.42	0.99	0.85–1.17	1.03	0.88–1.22
3	1.17	0.90–1.53	1.11	0.85–1.45	0.92	0.78–1.09	0.97	0.81–1.15
4	1.51	1.16–1.96	**1.44**	1.10–1.90	0.77	0.64–0.92	**0.82**	0.67–0.99
5 – Most deprived	1.69	1.25–2.27	**1.52**	1.11–2.07	0.72	0.57–0.90	**0.80**	0.63–0.99
Ethnicity								
White (ref)	–	–	–	–	–	–	–	–
Asian	1.99	1.17–3.22	**2.00**	1.15–3.32	0.69	0.41–1.09	0.65	0.39–1.04
Black	0.67	0.04–3.32	0.68	0.04–3.53	0.71	0.16–2.24	0.70	0.16–2.28
Mixed	0.43	0.10–1.16	0.39	0.09–1.08	1.10	0.63–1.85	1.21	0.68–2.06
Any other	1.43	0.22–**5**.21	1.01	0.15–3.82	1.90	0.64–5.27	2.60	0.84–7.49
Diagnosis								
Ulcerative colitis (ref)	–	–	–	–	–	–	–	–
Crohn’s disease	1.17	0.98–1.39	1.07	0.89–1.29	0.75	0.66–0.84	**0.82**	0.72–0.93
IBD-Control-8 score^c^								
Per unit increase	0.93	0.92–0.95	**0.96**	0.94–0.99	1.07	1.06–1.09	**1.06**	1.04–1.08
IBD-Control-VAS score^c^								
Per unit increase	0.99	0.99–0.99	(**0.99**)	(0.99–0.99)	1.01	1.01–1.02	(**1.01**)	(1.01–1.02)
PRO-2 remission status^c^								
Active (ref)	–	–	–	–	–	–	–	–
Remission	0.60	0.50–0.72	(**0.75**)	(0.61–0.92)	1.41	1.23–1.62	(**1.18**)	(1.02–1.37)
Comorbidities								
0 (Ref)	–	–	–	–	–	–	–	–
1	1.19	0.95–1.47	1.11	0.88–1.39	0.95	0.82–1.11	1.03	0.87–1.20
2+	1.87	1.36–2.53	**1.50**	1.06–2.09	0.76	0.58–0.99	**0.88**	0.65–1.16
Restriction to the home								
No (ref)	–	–	–	–	–	–	–	–
Yes	1.25	0.97–1.58	0.80	0.61–1.06	0.77	0.64–0.93	1.16	0.94–1.43
Mobility problems								
No (ref)	–	–	–	–	–	–	–	–
Yes	1.15	0.75–1.68	0.78	0.49–1.20	0.76	0.55–1.02	1.07	0.76–1.53
Support available								
No (ref)	–	–	–	–	–	–	–	–
Yes	0.79	0.62–1.01	0.82	0.64–1.06	1.11	0.93–1.33	1.09	0.91–1.32
Satisfied with IBD care								
Yes – completely (ref)	–	–	–	–	–	–	–	–
Yes – mostly	2.99	2.46–3.65	**2.80**	2.28–3.44	0.42	0.37–0.48	**0.47**	0.41–0.54
No – not at all	6.13	4.39–8.48	**5.02**	3.49–7.15	0.39	0.27–0.54	**0.53**	0.37–0.76

Univariate and multivariate binary logistic regression analyses.

Statistically significant ORs are in bold text.

aOR, adjusted odds ratio (multivariable analysis); 95% CI, 95% confidence interval; IBD, inflammatory bowel disease; OR, unadjusted odds ratio (univariate analysis); PRO-2, patient reported outcome-2; PROM, patient reported outcome measure; VAS, Visual Analogue Scale.

a In-person preference: Analyses focus on factors associated with expressing a ‘strong preference’ for in-person consultations (response = ‘never by telephone or video’ versus all other responses).

b Remote preference: Analyses focus on factors associated with expressing a ‘strong preference’ for remote consultations (response = ‘Mainly telephone or video’ versus all other responses).

c PROM scores: The adjusted ORs in the table are based on the multivariable model with IBD-Control-8 selected as the measure of current health status. Separate models were fitted for IBD-Control-VAS or PRO-2 remission status since both are strongly correlated with IBD-Control-8. Hence, the aORs for alternative PROMs are placed in parentheses (see section Results).

We focus on those factors identified as significant predictors of preference in multivariable models after adjusting for other factors.

### Demographic characteristics

Older age (>65 years versus ≤65 years) was positively associated with a strong in-person preference [adjusted OR (aOR): 1.59] and with a higher level of in-person preference (aOR: 1.45). There was a reciprocal negative association with a strong remote preference (aOR: 0.84). Males were significantly more likely than females to express a strong in-person preference (aOR: 1.59), but no significant associations with a higher level of in-person preference or with strong remote preference.

Findings for socioeconomic status were significant and consistent across all models, with a gradient of effects across the deprivation quintiles. Compared with those living in the least deprived areas (Q1), those in the most deprived areas (Q5) were significantly more likely to express a strong (aOR: 1.52) or higher level (aOR: 1.33) of in-person preference and were less likely to strongly prefer remote consultations in the future (aOR: 0.80).

In comparison with the majority of white respondents, those of Asian ethnicity were significantly more likely to express a strong (aOR: 2.0) or higher level (aOR: 1.71) of in-person preference. However, there was no significant association between ethnicity and a strong preference for mainly remote consultations.

### Comorbidities, restrictions to home, mobility, and support

People reporting two or more additional comorbidities were consistently more likely to prefer to be seen physically in the IBD clinic in the future, with an increased odds of strong (aOR: 1.50) or higher level (aOR: 1.28) of preference for in-person consultations. Multimorbidity was also associated with a significantly reduced likelihood of preferring ‘mostly remote’ consultations in the future (aOR: 0.88).

People reporting health problems restricting them to the home accounted for 13% of respondents (*n* = 752). We predicted that this factor may be associated with a preference for remote consultations, but we found no such association. Those reporting use of mobility aids accounted for 4% of our sample (*n* = 255), but this factor did not have any association with preferences. The survey also asked whether the respondent had someone they could rely upon for help, with 12% (*n* = 723) indicating they did not. However, the availability of support was not associated with consultation preferences in our survey sample.

### Inflammatory bowel disease subtype

In comparison to those with ulcerative colitis, patients with Crohn’s disease were significantly more likely to express a higher level of in-person preference (aOR: 1.20) and significantly less likely to express a strong remote preference (aOR: 0.82), although there was no difference with respect to strong in-person preference. Taken together, these results suggest at least some influence of disease subtype on preferences, with people having a diagnosis of Crohn’s disease being more likely to prefer physical (in-person) consultations after adjusting for other observed factors.

### Current health status

Each patient reported outcome measure (PROM) instrument (IBD-Control Questionnaire or PRO-2) was tested individually in different sets of models, since the health status scores are strongly correlated with each other.

*IBD-Control Questionnaire* – The IBD-Control-8 was used as the measure of current IBD status in the baseline models, and this score was consistently associated with consultation preference. For every unit increase in the IBD-Control-8 score (i.e. better control), the odds of strong in-person preference reduced by 4% (aOR: 0.96), the odds of being in a higher category of in-person preference reduced by 5% (aOR: 0.95), and the odds of a strong preference for remote increased by 6% (aOR: 1.06). Substituting the IBD-Control-VAS score as the health status measure showed similarly significant findings, with effects in the same direction (Table 2 and Supplementary Table S1, Supplemental digital content 1, https://links.lww.com/EJGH/B315).

We further investigated associations with the individual questions that comprise the IBD-Control-8 scale, replacing its total score with the component items. The distribution of IBD-Control-8 items responses across the three levels of preference are shown in Fig. 2. For all eight items, the proportion of people reporting the ‘negative’ response (i.e. worst option; scoring zero points) increased progressively from remote, through no preference to in-person preference, with statistically significant differences.

**Fig. 2.**
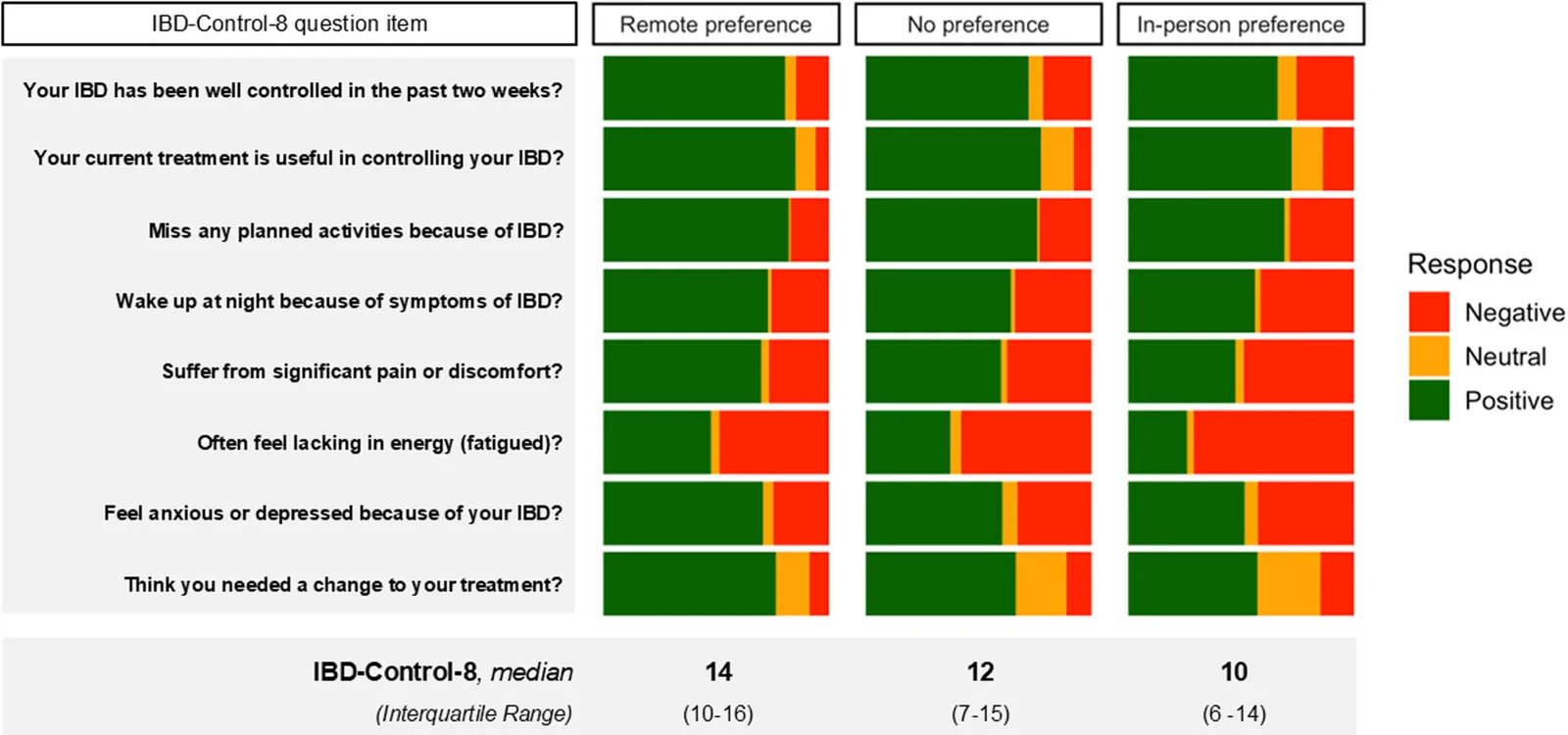
Distribution of responses to IBD-Control-8 questionnaire items according to current preference for future consultation among 5833 survey respondents. A negative response is assigned a score of zero in the IBD-Control-8 scale (i.e. the ‘worst’ response; red bars; which corresponds to ‘No’ for the first two questions and ‘Yes’ for the remaining six items). A positive response is assigned a score of two points (i.e. the ‘best’ response’; green bars; corresponding to ‘Yes’ for the first two questions and ‘No’ for the remaining six items). Neutral responses score one point (i.e. ‘intermediate’ response; amber bars; ‘Not sure’ for the item). The proportion of negative (‘worse’) responses was significantly higher among people with in-person preference for each of the eight items (*P* < 0.05 for all; *χ*^2^ test). Median scores for IBD-Control-8 were significantly lower (‘worse’) among those with in-person preference (*P* < 0.05; Kruskal–Wallis test). IBD, inflammatory bowel disease.

*PRO-2* – When PRO-2 remission status was used as the measure of health status, we found symptom-based remission was associated with a significantly *reduced* odds of strong (aOR: 0.75) or higher category (aOR: 0.67) of in-person preference and a reciprocal increase in odds of a strong remote preference (aOR: 1.18).

### Satisfaction with inflammatory bowel disease care

Fifty-four percent of respondents indicated they were satisfied with the care they had received for their condition (‘Completely’), 34% gave an intermediate rating (‘Mostly’), but 4% were dissatisfied (‘Not at all’). There was a strong association between reducing level of satisfaction and preference for in-person consultations with a gradient effect across the categories. Compared to those completely satisfied, those who were dissatisfied were significantly more likely to have a strong (aOR: 5.02) or higher level (aOR: 3.08) preference for in-person consultation and a much lower odds of remote preference (aOR: 0.53). The ORs for those who were ‘mostly’ satisfied were of smaller magnitude but in the same directions (Table 2 and Supplementary Table S1, Supplemental digital content 1, https://links.lww.com/EJGH/B315).

## Discussion

Most survey respondents with some prior experience of remote IBD consultations regarded such assessments as acceptable with almost two-thirds (64%) indicating no strong preference for any specific modality of appointment, and a further quarter (25.9%) preferring mostly remote consultations at the time of completing their survey. Nevertheless, one in 10 (9.7%) patients expressed a strong preference to avoid remote IBD consultations entirely in the future, answering ‘never by telephone or video’. Only a small proportion (5.1%) of our respondents reported experience of video consultations, suggesting telephone calls were the typical method for delivering telehealth within UK IBD services.

To our knowledge, this is the largest reported study of factors associated with patient preferences for modality of consultation among people living with IBD. A detailed survey of experiences of IBD telehealth in France has been reported among 300 respondents during the pandemic [17], but this did not specifically focus on patient-level factors associated with preferences. Some of our findings are consistent with factors observed previously in other conditions. Older people have been reported to be more likely to express in-person preference [8] and less likely to successfully complete a scheduled telephone appointment [9]. We also found that those living in more deprived areas had a greater tendency to favour physical consultations after adjusting for other factors. This is consistent with a recent systematic review of published studies of inequalities in uptake of remote consultations across secondary and tertiary care, whereby those with lower household income and lower educational attainment were least likely to successfully complete a remote consultation when it was offered [18].

We also observed that male patients were significantly more likely to express a strong preference for in-person IBD appointments than females. A study in orthopaedic practice suggested females may be slightly more inclined to express a preference for remote consultations than males [10], and the general literature suggests females are generally more likely to complete a remote consultation when invited [18]. Our observation of increased likelihood of in-person preference among those of Asian ethnicity is interesting. Non-White ethnicities have been reported to be less likely to complete a remote secondary/tertiary care consultation in studies based in English-speaking countries (USA, Canada, and Australia) where White ethnicity is the majority [18]. While reduced uptake of remote services has been attributed partly to language barriers in these studies, our survey respondents had successfully completed a detailed online survey in English, and the findings were specific to those of Asian ethnicity. There may be specific cultural factors linked to ethnicity that influence a preference for physical consultations.

Interestingly, we demonstrated no increase in the odds of remote appointment preference among those using mobility aids or reporting a condition that usually limits movement outside the home, consistent with a recent scoping review of preferences among those living with disabilities [19]. We also found that those with a higher number of comorbidities had an *increased* tendency towards in-person preference. It is clearly wrong to assume that those with poor mobility or multimorbidity would automatically favour telehealth solutions.

Despite the expansion of remote services, published studies of consultation preferences expressed by people living with IBD remain quite limited. A recent single-centre randomised study administered postappointment surveys (*n* = 86) following randomisation of adults to either in-person, telephone, or video consultation, reporting statistically higher overall patient preference (10-point rating scale) for in-person versus the telephone modality (9.1 versus 7.8; *P* = 0.014) [20]. This study elicited modality preferences and ratings for certain aspects of appointments. The highest rated factors among those preferring in-person visits were ‘optimal communication and interaction’, whereas people with remote preference rated ‘time and cost savings’ most highly. Another single-centre study from a tertiary care centre examined factors associated with ‘failure’ of scheduled telephone or video consultations, reporting no significant associations with demographics (age, sex, ethnicity) or insurance status for failed telephone appointments [21]. A survey of 141 IBD patients who had their appointment changed from in-person to telephone during the COVID-19 pandemic found that lower levels of satisfaction were associated with more active disease in one service in Ireland [22]. Only 55% of 300 patients in the French survey reported that care quality was the same via telemedicine compared with face-to-face, and most felt it was not an appropriate modality for a ‘flare-up’ consultations [17].

Our observation of a somewhat increased in-person preference among those with Crohn’s disease (compared with UC) has not been reported previously. This is consistent with the possibility that in-person consultations may be favoured more often in those with Crohn’s disease because physical examination is perceived to play a greater role in assessment. A multicentre study of consultation preferences in those with neuromuscular conditions found that 40% of patients expressed concerns about the lack of physical examination during remote assessments, and such patients had significantly higher odds of selecting in-person visits [12]. Similar concerns have been reported in other conditions [11].

Current health status, as reflected by IBD-specific PROM scores, was highly associated with future visit preferences. Regardless of the choice of PROM instrument (IBD-Control-8, IBD-Control-VAS, or PRO-2), a strong remote preference was associated with better control or symptom-based remission, whereas in-person preference was associated with worse control or active disease. This is consistent with the idea that remote care may be favoured for ‘routine visits’ (i.e. when patients are feeling well or stable), whereas in-person visits may be favoured by those with more acute or active problems, as has been observed in a general population survey asking about modality preference across different clinical scenarios [23].

Reassuringly, less than 5% of IBD patients in our sample reported a significant level of dissatisfaction with care received during the pandemic – suggesting UK services mostly provided good care during this period. However, we observe a very strong association with dissatisfaction and in-person preference. Dissatisfied patients may feel that remote consultations offer a less comprehensive assessment, or provide fewer opportunities to discuss and resolve any perceived shortcomings in past care.

The extent to which UK IBD services routinely elicit, record, and act upon preferences for modality of appointment is largely unknown. Scheduling of the next clinic appointment is often decided at the time of a consultation, and yet our findings with respect to current disease control suggest preferences are likely to depend on the subsequent trajectory of health status. Ideally, services would have flexibility in clinic capacity and administration to accommodate varying preferences while maintaining equitable access to specialist advice. Although the impact of the pandemic has receded, some services may have retained a legacy of remote options as the norm for specific clinics. Moreover, telephone-based ‘flare lines’ are often the first-line option for people needing to access urgent assessment between routine appointments. Our findings suggest that greater consideration may be needed for one in 10 people who find remote telephone interactions unacceptable. Research is needed to determine whether such patients experience inequalities or poorer outcomes in settings where key components of IBD services have evolved into mainly remote offerings. Clinics should be designed and organised to ensure those unwilling or unable to engage with remote care models are not disadvantaged, because the push towards telehealth has the potential to exacerbate existing inequalities linked to patient characteristics in IBD care [24].

Strengths of our study include the very large sample size with UK-wide coverage and the inclusion of questions covering a range of putative patient-level predictors, including both ‘fixed’ characteristics and more ‘dynamic’ factors such as current symptoms, disease-specific QoL, and satisfaction with IBD care. Our use of alternative modelling approaches for exploring influential factors yielded consistent findings, which provides additional confidence in our findings. The binary logistic models sought associations with ‘strong’ preferences, whilst the OLR models sought to validate these findings and mitigate potential ‘loss of information’ from using dichotomised outcomes.

Our study has a number of limitations. First, participants were originally recruited via an online tool, and study findings may not be generalisable to people who have difficulty accessing or using internet-enabled devices. Digital exclusion is reported to be associated with lower socioeconomic status [25], but our online survey included 592 people (10%) living in areas classified as the most deprived quintile of the UK. Nevertheless, we accept that our findings from this survey may not be applicable to the entire IBD population. Second, as with most research in this field, our study was cross-sectional and did not examine variations in preferences over time. We did include some ‘dynamic’ predictors subject to temporal variation (i.e. current health status, current satisfaction with care) and demonstrated strong associations with these variables and current consultation preference. These factors are likely to be potential drivers for changes in preferences over time.

Third, despite having survey information for a range of potential predictors, there are likely to be many more unobserved factors that might influence preference for remote or in-person consultations. As well as individual-level factors, there will be service-level influences that reflect the way in which remote consultations are delivered and the quality of that experience (e.g. local support and information provided for remote consultations, range of technologies offered, staff training and competencies). Relatedly, there may be variations in the quality of remote care operating at the level of individual clinicians that could impact on an individual patient’s preferences. It was not possible to study service-level or clinician-level factors in this secondary analysis of a large-scale survey but these additional factors could be investigated in future research. However, our findings in relation to care satisfaction may partly reflect service-level factors, since those with a poor experience of recent care were most likely to prefer in-person appointments in the future.

As pandemic disruptions continue to recede, services may have retained a legacy of ‘remote-only’ options for specific components of services. Teams may wish to review local processes to ensure that those who opt-out of remote visits (or have failed remote encounters, Did-Not-Attends) are not unfairly disadvantaged. Clinics should be designed and organised to ensure that such patients do not experience delays compared to those willing or able to engage with remote care models. Our findings suggest that specific consideration is needed for the 10% of people who find remote interactions unacceptable. Research is needed to determine whether such patients experience poorer outcomes in contexts where services have prioritised telehealth as the primary mode of delivering care.

The obvious solution for assigning the most suitable modality for a patient’s next appointment is to simply ask about preference and then provide the preferred option. However, in the UK NHS context, scheduling of the next clinic appointment is often decided at the end of a consultation. Our findings with respect to disease control suggest preferences may change depending on the subsequent trajectory of health status. The extent to which IBD services routinely elicit, record, and act upon service user preferences for modality of appointment is unknown. It is unclear whether all IBD services have the flexibility of capacity and appointment administration to accommodate differing or changing preferences whilst maintaining equitable access to specialist advice and appropriate waiting times for all. Future research and quality improvement efforts may benefit from a focus on variations in local models of remote care delivery between centres and their associations with care costs, outcomes, and patient experience.

## Acknowledgements

None.

### Conflicts of interest

There are no conflicts of interest.

## Supplementary Material
