# Supplementary material for: Factors associated with preference for in-person versus remote consultations in inflammatory bowel disease in the United Kingdom: secondary analysis of an online survey
Source: Eur J Gastroenterol Hepatol. 2026 Jun 4;38(11):1290–8. doi: 10.1097/MEG.0000000000003220 (PMC13626586; doi:10.1097/MEG.0000000000003220)
Supplement: Supplementary file 1 [file ejgh-38-1290-s001.docx]

**SUPPLEMENTARY MATERIALS**

**Supplementary Table S1: Factors associated with a higher level of preference for in-person consultations among 5,833 survey respondents with prior experience of both in-person and remote consultations:** Univariate and multivariable ordinal logistic regression analyses

| Characteristic | In-person preference^1^  (*Remote🡪 None🡪 In-person*) | | | |
| --- | --- | --- | --- | --- |
|  | OR | 95% CI | aOR | 95% CI |
|  |  |  |  |  |
| Age group |  |  |  |  |
| ≤65 Years (ref) | — | — | — | — |
| >65 Years | **1.26** | 1.09-1.47 | **1.45** | 1.24-1.71 |
| Gender |  |  |  |  |
| Female (ref) |  |  |  |  |
| Male | 0.95 | 0.85-1.06 | 1.05 | 0.93-1.17 |
| Deprivation quintile |  |  |  |  |
| 1 - Least deprived (ref) | — | — | — | — |
| 2 | 1.04 | 0.90-1.20 | 0.99 | 0.86-1.15 |
| 3 | 1.10 | 0.95-1.29 | 1.05 | 0.90-1.23 |
| 4 | **1.36** | 1.15-1.60 | **1.29** | 1.09-1.52 |
| 5 - Most deprived | **1.48** | 1.21-1.80 | **1.33** | 1.09-1.63 |
| Ethnicity |  |  |  |  |
| White (ref) | — | — | — | — |
| Asian | **1.69** | 1.13-2.53 | **1.71** | 1.15-2.56 |
| Black | 1.14 | 0.42-3.21 | 1.07 | 0.38-3.06 |
| Mixed | 0.80 | 0.50-1.29 | 0.70 | 0.43-1.13 |
| Any other | 0.65 | 0.23-1.89 | 0.48 | 0.16-1.44 |
| Diagnosis |  |  |  |  |
| Ulcerative colitis (ref) | — | — | — | — |
| Crohn's disease | **1.29** | 1.16-1.44 | **1.20** | 1.08-1.34 |
| IBD-Control-8 score^2^ |  |  |  |  |
| Per unit increase | **0.93** | 0.92-0.94 | **0.95** | 0.94-0.97 |
| IBD-Control-VAS score^2^ |  |  |  |  |
| Per unit increase | **0.99** | 0.99-0.99 | n/a | n/a |
| PRO2 remission status^2^ |  |  |  |  |
| Active (ref) | — | — | — | — |
| Remission | **0.67** | 0.59-0.76 | n/a | n/a |
| Comorbidities |  |  |  |  |
| 0 (ref) | — | — | — | — |
| 1 | 1.09 | 0.95-1.24 | 1.02 | 0.88-1.17 |
| 2+ | **1.51** | 1.20-1.91 | **1.28** | 1.00-1.64 |
| Restriction to the home |  |  |  |  |
| No (ref) | — | — | — | — |
| Yes | **1.28** | 1.09-1.50 | 0.85 | 0.71-1.02 |
| Mobilty problems |  |  |  |  |
| No (ref) | — | — | — | — |
| Yes | 1.26 | 0.97-1.63 | 0.90 | 0.68-1.19 |
| Support available |  |  |  |  |
| No (ref) | — | — | — | — |
| Yes | 0.86 | 0.73-1.01 | 0.89 | 0.76-1.05 |
| Satisfied with IBD care |  |  |  |  |
| Yes - completely (ref) | — | — | — | — |
| Yes - mostly | **2.51** | 2.23-2.82 | **2.27** | 2.01-2.57 |
| No - not at all | **4.09** | 3.07-5.43 | **3.08** | 2.29-4.14 |

OR=unadjusted odds ratio (univariate analysis); aOR=adjusted odds ratio (multivariable analysis); 95% CI=95 percent confidence interval. Statistically significant ORs are in **bold text**.

**^1^ In-person preference**: Analyses focus on factors associated with an increasing degree of in-person preference across the spectrum from remote preference, through no preference to in-person preference. Odds ratios reflect the odds of being in a “higher” category. See **Methods**.

**^2^** **PROM scores:** The adjusted ORs in the table are based on a multivariable model that included IBD-Control-8 as the IBD-specific PROM. Hence, only univariate odds ratios are shown for IBD-Control-VAS and PRO2 remission. Both are strongly correlated with IBD-Control-8 and therefore omitted from multivariable model.. See results section for further details of associations for each PROM score and components.

**Supplementary Table S2: Summary of associations between patient characteristics and preference for future modality of IBD consultations among 5,833 people with IBD across the UK**

|  | **In-person preference^1^**  (Vs Not) | **Level of in-person preference^2^** | **Remote**  **Preference^1^**  (Vs Not) |
| --- | --- | --- | --- |
| **Older age** (≥65 years) | ↑ | ↑ | ↓ |
| **Male gender** | ↑ | ns | ns |
| **Living in deprived area** | ↑ | ↑ | ↓ |
| **Asian ethnicity** (vs White) | ↑ | ↑ | ns |
| **CD** (vs UC) | ns | ↑ | ↓ |
| **IBD-Control-8** (better control) | ↓ | ↓ | ↑ |
| **PRO-2 Remission** | ↓ | ↓ | ↑ |
| **≥2 Comorbidities** | ↑ | ↑ | ↓ |
| **Dissatisfaction with care** | ↑ | ↑ | ↓ |

1. See **Table 2** of main manuscript for relevant Binary Logistic Regression models.

2. See **Supplementary Table S1** for relevant Ordinal Logistic Regression models.

**Appendix-1	Questionnaire items and response options**

| **Question item** | **Response options** |
| --- | --- |
| **Demographics** |  |
| What is your date of birth? | Date |
| What is your gender? | Female \| Male \| Other \| Prefer not to say |
| Which of the following best describes your ethnicity? | WB, White: British \| WI, White: Irish \| WG, White: Gypsy or Irish Traveller \| WO, White: Other \| BC, Black: Caribbean \| BA, Black: African \| BO, Black: Other \| AI, Asian: Indian \| AP, Asian: Pakistani \| AB, Asian: Bangladeshi \| AC, Asian: Chinese \| AO, Asian: Other \| MWBC, Mixed: White & Black Caribbean \| MWBA, Mixed: White & Black African \| MWA, Mixed: White & Asian \| MO, Mixed: Other \| OA, Other: Arab \| OO, Any other ethnicity |
| **Diagnosis** |  |
| What type of inflammatory bowel disease do you have? | CD, Crohn's disease \| UC, Ulcerative colitis \| IBDU, Inflammatory bowel disease unclassified \| Other, Other |
| **Comorbidities** |  |
| - Do you have hypertension (high blood pressure) requiring medication? - Do you have diabetes (e.g. type 1 or type 2 diabetes) for which you take insulin or tablets? - Do you have a respiratory or chest disease which has an impact on your day-to-day life (asthma where you take inhalers or tablets every day; emphysema or COPD which limits how much you can do)? - Have you ever been diagnosed with angina (chest pain caused by your heart), a heart attack or stroke? - Do you have a diagnosis of heart failure which limits how much you can do? - Have you been diagnosed with heart valve disease which limits how much you can do or has required valve surgery? - Do you have chronic kidney disease? | Yes \| No |
| **Restrictions, support available and mobility** |  |
| In general, do you have any health problems that require you to stay at home? | Yes \| No |
| If you need help, can you count on someone close to you? | Yes \| No |
| Do you regularly use a stick, walking frame or wheelchair to get about? | Yes \| No |
| **Current health status** |  |
| **IBD-Control-8 Questionnaire**   - Do you believe that your IBD has been well controlled in the past two weeks? - Do you believe that your current treatment is useful in controlling your IBD? - In the past 2 weeks did you miss any planned activities because of your IBD ? (e.g. attending school/college, going to work or a social event) - In the past 2 weeks, did you wake up at night because of symptoms of IBD ? - In the past 2 weeks did you suffer from significant pain or discomfort? - In the past 2 weeks did you often feel lacking in energy (fatigued)? (by 'often' we mean more than half of the time) - In the past 2 weeks did you feel anxious or depressed because of your IBD? - In the past 2 weeks did you think you need a change in your treatment? | Yes \| No \| Not sure |
| - How would you rate the OVERALL control of your IBD in the past two weeks? | Visual Analogue Scale (0-100) |
| **PRO-2 Symptoms**   - How many liquid or soft stools did you pass on the following days? - How would you rate your abdominal pain over the past seven days? - On average, how many times a day would you open your bowels when you are well? - How many times are you currently opening your bowels each day? (By currently we mean over the past 3 days) - Are you currently passing blood in your stool? (By currently we mean over the past 3 days) | Number for each of last seven days  None \| Mild \| Moderate \| Severe  Number  Number  None \| Blood streaks seen in the stool \| Blood in most stools \| Only blood passed |
| **Prior remote consultations** |  |
| Since the COVID-19 lockdown, have you had an appointment for any of the following services? (Please tick all that apply) | A telephone consultation with an IBD doctor \| A video consultation with an IBD doctor \| A telephone consultation with an IBD nurse \| A video consultation with an IBD nurse \| None of the above |
| Was this the first time you have had a telephone or video review with an IBD doctor or nurse? | Yes \| No, I had a telephone or video review before the COVID pandemic \| Unsure/cannot remember |
| **Current preference for future consultations** |  |
| What would be your preference for IBD appointments in the future? | Mostly by telephone or video \| Sometimes by telephone or video \| Never by telephone or video \| I do not mind |
| **Experience rating of IBD care** |  |
| In your opinion, have you received the care and treatment you needed for your IBD during the pandemic? | Yes, completely \| Yes, mostly \| No, not at all \| I did not need any care or treatment during this time \| Unsure/cannot remember |
